# Antimicrobial Properties of Chilean Native Plants: Future Aspects in Their Application in the Food Industry

**DOI:** 10.3390/foods11121763

**Published:** 2022-06-15

**Authors:** María Carolina Otero, Juan A. Fuentes, Cristian Atala, Sara Cuadros-Orellana, Camila Fuentes, Felipe Gordillo-Fuenzalida

**Affiliations:** 1 Escuela de Química y Farmacia, Facultad de Medicina, Universidad Andrés Bello, República 252, Santiago 8320000, Chile; maria.otero@unab.cl; 2 Laboratorio de Genética y Patogénesis Bacteriana, Departamento de Ciencias Biológicas, Facultad de Ciencias de la Vida, Universidad Andrés Bello, Santiago 8320000, Chile; jfuentes@unab.cl; 3 Instituto de Biología, Facultad de Ciencias, Pontificia Universidad Católica de Valparaíso, Campus Curauma, Avenida Universidad 330, Valparaíso 2340000, Chile; cristian.atala@pucv.cl; 4 Laboratorio de Microbiología Aplicada, Centro de Biotecnología de los Recursos Naturales, Facultad de Ciencias Agrarias y Forestales, Universidad Católica del Maule, Avda. San Miguel 3605, Talca 3480112, Chile; scuadros@ucm.cl (S.C.-O.); camila.fuentes@alu.ucm.cl (C.F.)

**Keywords:** metabolites, active packaging, microorganisms

## Abstract

Food contamination with microorganisms is responsible for food spoilage, deterioration and change of organoleptic properties of foods. Besides, the growth of pathogenic microorganisms can provoke serious health problems if food is consumed. Innovative packaging, such as active packaging, is increasing rapidly in the food industry, especially in applying antimicrobials into delivery systems, such as sachets. Chile is a relevant hotspot for biodiversity conservation and a source of unique bio-resources with antimicrobial potential. In this review, fifteen native plants with antimicrobial properties are described. Their antimicrobial effects include an effect against human pathogens. Considering the emergence of antimicrobial resistance, searching for new antimicrobials to design new strategies for food pathogen control is necessary. Chilean flora is a promising source of antimicrobials to be used in active packaging. However, further studies are required to advance from laboratory tests of their antimicrobial effects to their possible effects and uses in active films.

## 1. Introduction

Microorganisms are the most common cause of deterioration in the food industry, manifesting as changes in texture, odors, and unpleasant flavors due to metabolic activity [1]. Microorganisms can proliferate in the food, bind to other matrices such as polymers like polyethylene, wood, glass, polypropylene, rubber, etc., and develop other forms of organization such as biofilms [2]. Pathogens such as *Bacillus cereus, Escherichia coli, Listeria monocytogenes, Helicobacter pylori, Salmonella enterica,* and *Staphylococcus aureus* can form biofilms, and once they contaminate food, they can cause severe diseases in humans [3]. Those microorganisms could reach the food or food packages by a lack of correct disinfection or contamination of the food processing chain.

These microorganisms have mechanisms of resistance to common chemicals due to their constant use [4]. Thus, identifying resistance in different strains would help to prevent and reduce food spoilage [5]. Among the main mechanisms of resistance developed by microorganisms, we can find reduced cell membrane permeability, expulsion pumps, mutation of target sites, and enzymatic hydrolysis [6]. At first, the quantity and size of proteins in the membrane’s pores can be selectively variable, decreasing the permeability of external agents’ entry [7]. An expulsion or efflux pump is a self-protection mechanism in the membrane of Gram-negative bacteria where unwanted substances are eliminated from the cell; however, it is not a specific system [8]. The mechanism of enzymatic hydrolysis consists of the production of hydrolase and inactivation by hydrolysis or modifying the foreign substance, achieving optimal resistance [9]. Finally, some groups of bacteria can evidence changes in their binding site, making them inefficient or exhibiting reduced affinity to certain substances, reducing their effect [10]. However, bacteria such as *Campylobacter* spp., *Salmonella* spp., and *Enterococcus* spp. have variable resistance mechanisms conferring superior properties [11,12,13]. Fortunately, technology and innovation are increasing rapidly in the food industry, especially in new designs of delivery systems for bioactive compounds [14].

Active packaging (AP) refers to any packaging in which active components have been deliberately included to improve the food shelf life, expanding the protection function of the container [15]. AP systems are composed of sachets (emitters) and pads (absorbers) placed inside packages, where active ingredients are incorporated. These compounds interact directly or indirectly with food to prevent or retard the growth of microorganisms [15,16,17]. In addition, pads remove unwanted compounds from the food or its environment, for example, moisture, carbon dioxide, oxygen, ethylene, or odor. Meanwhile, sachets add compounds to the packaged food or headspace, such as antimicrobial compounds, carbon dioxide, antioxidants, flavors, ethylene, or ethanol [17,18].

Chilean native flora is interesting since it is highly endemic despite its relatively low diversity [19]. Nearly 40% of Chile’s registered vascular plant species are endemic to the country, and a similar proportion is native, usually shared with Peru, Bolivia, and/or Argentina [20]. Native and endemic vascular plant species add up to over 80% of total vascular plant diversity, making Chile a relevant hotspot for biodiversity conservation and a source of unique bio-resources [21]. Endemic species, such as *Peumus boldus* and *Quillaja saponaria*, have been highly studied regarding their components and biological properties of extracts and purified metabolites [22]. However, other plants commonly consumed by native indigenous people or rural populations due to their known effects on human health have been less scientifically examined. Furthermore, innovation with novel antimicrobial compounds, such as those found in native flora, provides new properties that allow better and safer conservation for the food industry [23]. Hence, the objective of this work was to describe the antimicrobial properties found in Chilean endemic plants to propose future uses in the application of AP systems for the food industry.

## 2. Materials and Methods

The review was performed based on analyzing scientific data published about native Chilean plants and their antimicrobial effects. The information was gathered using the NCBI-Pubmed, Google Scholar, and Mendeley databases using the words: “Chilean plant” and “antimicrobial properties/activities” (i.e., *Peumus boldus*, antimicrobial properties/activities). Scientific literature from 1992 to 2022 was used for this work.

## 3. Antimicrobial Properties of Chilean Native Plants

### 3.1. Acaena magallánica

*Acaena magellanica* (Lam.) Vahl is a widely distributed perennial herb of 14 cm in height from the Rosaceae family, native to Argentina and Chile [20]. In Chile, it is usually found throughout the whole length of the country, from sea level to high elevations [24]. Its zoocoric fruits are easily entangled and dispersed by animals. Other morphologically similar *Acaena* species in Chile are also used for medicinal purposes as *A. magellanica* [20,21].

Air-dried plant material was extracted sequentially with hexane, dichloromethane, and methanol. Using the agar dilution method for antimicrobial testing, hexane and dichloromethane extracts presented minimal inhibitory concentrations (MIC) of 1000 μg/mL against the clinical isolate, *Escherichia coli* ATCC 25922, and methanol extracts showed an MIC of 1000 μg/mL against the pathogenic fungi *Microsporum canis* C112 [25].

Air-dried ground aerial parts of the plant were used to prepare different extracts. From hexane extraction, β-sitosterol was obtained, and from a dichloromethane extract, oleanolic acid was obtained. From methanolic extract, rhamnetin, quercetin, saponin, quercetin 3-*O*-β-D-galactoside, quercetin 3-*O*-β-D-glucoside and ellagic acid were identified. Finally, catechin and the saponins tormentic acid-28-*O*-β-D-galactopyranoside, and 28-*O*-β-D-glucopyranoside, were isolated using methanol/water [26].

### 3.2. Aristotela chilensis

*Aristotelia chilensis* (Mol.) Stuntz, from the Elaeocarpaceae family, is a 4–6 m shrub or small tree with yellow-white flowers and edible black-colored fruits, which naturally grows in central and southern Chile and southwestern Argentina [20]. Fruits are rich in antioxidants and vitamin C [27].

Lyophilized samples of berry fruits were extracted in an acidified methanol/water solution, and the polyphenolic profile was determined. Sample contained delphinidin-3-sambubioside-5-glucoside, delphinidin-3,5-diglucoside, cyanidin-3-sambubioside-5-glucoside, cyanidin-3,5-diglucoside, delphinidin-3-sambubioside, delphinidin-3-glucoside, cyanidin-3-sambubioside, cyanidin-3-glucoside, myricetin-3-galactoside, myricetin-3-glucoside, quercetin-galloyl-hexoside, rutin, ellagic acid, quercetin-3-galactoside, quercetin-3-glucoside, dimethoxy-quercetin, myricetin and quercetin [28]. In that work, a colorimetric broth microdilution method was used to determine the MIC and the minimum bactericidal concentration (MBC) of the extract on *Listeria innocua* CECT 910, *Serratia marcescens* CECT 854, *Aeromonas hydrophila* CECT 5734, *Achromobacter denitrificans* CECT 449, *Alcaligenes faecalis* CECT 145, *Enterobacter gergoviae* CECT 857, *Enterobacter amnigenus* CECT 4078 and *Shewanella putrefaciens* CECT 5346, obtaining values between 50–80 and 50–100 g/L, respectively [28].

Using a thin-layer chromatographic (TLC) agar overlay protocol, it was determined a minimal inhibitory amount (MIA) against *E. coli* EDL 933 (ATCC 700927), *Staphylococcus aureus* (ATCC 6538), and *Bacillus subtilis* (ATCC 6633) of different leave extracts (dichloromethane/methanol, aqueous or ethanol). In addition, a MIA lower than 0.5 mg was observed for *S*. *aureus* using the dichloromethane/methanol extract [29].

### 3.3. Azorella acaulis

*Azorella acaulis* is a yellowish-green compact resinous cushion shrub from the Apiaceae family that grows at high elevations in the Andes of Chile and Argentina [20]. There are over twenty five species of *Azorella* in Chile, some of which have similar medicinal properties [20,30].

Using petroleum ether extract from the whole plant, it was possible to isolate tetracyclic diterpenoids azorenallol, 7-deacetylazorellanol, and 13-hydroxy-7-oxoazorellane, which were reported to present activity against the flagellated protozoan *Trichomonas vaginalis* (Ant-1 strain) [31].

Diterpenoids isolated from *Azorella compacta*, *Azorella madreporica*, *Mulinum crassifolium*, and *Azorella acaulis* have shown anti-tuberculosis activity by affecting the growth of *Mycobacterium tuberculosis* H37Rv ATTC 27294 strain, an antibiotic susceptible strain and strain *Mycobacterium tuberculosis* CIBIN/UMF15:99, a resistant strain. Using a microplate blue assay, the diterpenoids 13-β-hydroxyazorellane, azorellanol, 17-acetoxy-13-α-hydroxyazorellane, 7-deacetyl-azorellanol, azorellanone, 13-epiazorellanol, yaretol, 13-hydroxy-mulin-11-en-20-oic acid, mulin-11,13-dien-20-oic acid, 13,14-dihydroxy-mulin-11-en-20-oic acid, mulinic acid, 17-acetoxy-mulinic acid, 13,20-dihydroxymulin-11-en, and mulinenic acid, had MIC ranging between 12.5–100 μg/mL. Compounds azorellanol and 7-acetoxy-13-α-hydroxyazorellane had the lowest values of MIC (12.5 μg/mL) for both strains [32].

### 3.4. Berberis microphylla

*Berberis microphylla* is a thorny shrub from the Berberidaceae family native to southern Argentina and Chile [20]. Its edible blue–black berries are harvested for jams and liquors but are also eaten fresh. It is grown commercially at a small scale for its fruits and medicinal uses. Fruits are rich in antioxidants, and the plant is known for its antimicrobial properties [33,34].

Fruit metabolite extracts were prepared using methanol/water. From them, the following compounds were isolated: delphinidin-3,7-β-*O*-diglucoside, petunidin-3,7-β-*O*-diglucoside, malvidin-3,7-β-*O*-diglucoside, peonidin-3,7-β-*O*-diglucoside, cyanidin-3,7-β-*O*-diglucoside, 3- and 4-*trans*-caffeoyl-glucaric acids and possibly 2- or 5-*trans*-caffeoyl-glucaric acids [35]. Methanolic extracts from leaf, stem, and root were separated through high performance liquid chromatography and mass spectrometry (HPLC-MS), identifying eleven alkaloids (allocryptopine, berberine, calafatine, isocorydine, jatrorrhizine, palmatine, protopine, reticuline, scoureline, tetrahydroberberine, and thalifendine), whose distribution was tissue- and zone-dependent [36].

Using inhibition zone diameters measurements, leaf, stem, and root alkaloid methanolic extracts presented antimicrobial activity, dependent on quantity used, against the Gram-positive bacteria *B. cereus* ATCC 11778, *S. aureus* ATCC 25923, *S. epidermidis* ATCC 12228 and *B. subtilis* ATCC 6633 [28]. A 2000-μg/disc of the root extract had the most significant inhibition zone compared to a similar amount of stem and leave extracts. A comparable response was observed with 2000 μg/disc of berberine, one of the main isoquinole alkaloids of the root extract, against *S. aureus* and *S. epidermidis* [37]. In addition, the MIC and the MBC were determined for the bacteria mentioned above, where the stem and root alkaloid extracts showed the lower MIC and MBC values for *S. epidermidis* (83 and 167 μg/mL, respectively) [37].

In other work, MIC against *S. aureus* 4222, a *norA* mutant *S. aureus* strain KLE 820 (mutant in a multidrug resistance pump), and the bacteria *E. coli* were analyzed. These bacteria were treated with berberine, a solution of boiled commercial *B. microphylla* tea, a solution of in vivo shoot of the plant (dried material), and media derived from shoot cultures incubated with different plant growth regulators (thidiazuron, picloram, and jasmonic acid) [38]. In this assay, culture media from shoots pretreated with the plant growth regulator, jasmonic acid, had the highest MIC (0.248 μg/mL for all the tested strains) [38]. These results suggest an effect of the hormone on antimicrobial production.

Methanolic/water extracts of the whole plant showed an half-maximal inhibitory concentration (IC_50_) of 38.4 μg/mL in a 3-(4,5-dimethylthiazol-2-yl)-2,5-diphenyltetrazolium bromide viability test against *Trypanosoma cruzi*, the etiologic agent of Chagas disease [39].

### 3.5. Buddleja globosa

*Buddleja globosa* Hope is a relatively tall shrub from the Scrophulariaceae family (previously in Buddlejaceae) endemic to Chile [20]. It has orange globose heads that are characteristic of the species. Its leaves are widely used in ancestral medicine due to their known topical wound- and burn-healing effects and for treating gastric ulcers [33]. It is also used in other countries as an ornamental plant and can form hybrids with *B. davidii* [40].

Ethanolic extracts of leaves were separated using TLC. Fractions were evaluated for bacteriostatic activity against *S. aureus* ATCC 25923 and *E. coli* ATCC 25922 using an agar well diffusion assay. Further purification revealed that the molecule responsible for this effect was verbascoside, presenting a MIC of 1 mM [41].

Essential oils (EOs) have been extracted using hydrodistillation and presented monoterpenes (α-pinene, camphene, sabinene, β-pinene, myrcene, *p*-cymene, limonene, and γ-terpinene), oxygenated monoterpenes (1,8-cineole, *cis*-sabinene hydrate, linanool, *cis*-thujone, *trans*-thujone, *cis*-*p*-mentha-2,8-dien-1-ol, *trans*-sarbinol, camphor, thujanol, citronellal, *cis*-chrysanthenol, borneol, terpinen-4-ol, thuj-3-en-10-al, α-terpineol, dihydro carveol, *trans*-carveol, carvone, thymol, and carvacrol), sesquiterpenes hydrocarbons (α-humulene, γ-gurjunene, γ-cadinene, δ-cadinene and *(E)*-caryophyllene), oxygenated sesquiterpenes (elemol, spathulenol, caryophyllene oxide, β-cedrene epoxide, caryophylla-4(12), 8(13)-dien-5-ol, murrolol, and bulnesol) and other volatiles (sabina ketone and *ar*-curcumene), where monoterpenes were the most abundant [42]. In addition, EOs demonstrated an antimicrobial activity and a disruptive effect in the membrane of the Gram-positive spore-forming pathogen *Paenibacillus larvae*, a causative agent of the American foulbrood, one of the most destructive brood diseases in *Apis mellifera*. Antimicrobial activity, measured by the broth microdilution method, was correlated with the presence of pulegone, carvone, (Z)-β-ocimene, δ-cadinene, camphene, terpinen-4-ol, elemol, β-pinene, β-elemene, γ-cadinene, α-terpineol, and bornyl acetate. MIC determined for EOs against *P. larvae* was 25 μg/mL [42].

The agar dilution method was used to test the antimicrobial activity of the stem bark CHCl_3_ extract. The extract exhibited antifungal activity against dermatophytic fungal species *Trichophyton rubrum*, *Trichophyton interdigitale,* and *Epidermophyton floccosum* at 250 μg/mL. Further purification of extracts revealed that molecules responsible for the antimicrobial effect were diterpene buddlejone, the bisditerpene maytenone, the sesquiterpenes buddledin A and buddledin B, and the diterpene deoxybuddlejone, where buddledin A and B were the most effective metabolites with MIC values of 43 and 51 µM, respectively, against the microorganisms mentioned above [43].

Petroleum ether extracts and later extraction with methanol or water showed inhibition of [^3^H]-hypoxanthine uptake in a resistant strain of protozoan parasite *Plasmodium falciparum* strain K1 with an IC_50_ of 6.2 μg/mL and 8.7 μg/mL, respectively [44].

### 3.6. Cryptocarya alba

*Cryptocarya alba* (Mol) Looser is a native Chilean tree from the Lauraceae family. It is also present in Argentina and is found in Chile from Coquimbo to the Araucania region, growing from sea level up to 1500 m [20]. The fruit is edible (cooked or fresh), and the aromatic leaves can be used as a facial mask to clean the skin [33]. In addition, the EOs of this specie seem to have antioxidant and antibacterial properties [45].

Fruits of *C. alba* were extracted with MeOH and analyzed by TLC, obtaining the compound cryptofolione and a cryptofolione derivative 6-(4,6-dimethoxy-8-phenyl-octa-1,7-dienyl)-4-hydroxy-tetrahydro-pyran-2-one [46]. Aerial parts were used to prepare EOs by water distillation. A sample was analyzed by gas chromatography and mass spectrometry (GC-MS), detecting around fourteen compounds where the most abundant were α-terpineol and eucalyptol [45].

The agar disk diffusion assay was used to examinates the antimicrobial activity of EOs and purified compounds against *H. pylori, S. aureus, E. coli,* and yeast *Candida albicans*. The compound α-terpineol had a MIC of 16 μg/mL against the clinical isolates *E. coli* and *C. albicans* [45]. Cryptofoline isolated from fruit extracts had an anti-*T. cruzi* and *Leishmania* spp. effect [46].

### 3.7. Fragaria chiloensis spp. chiloensis

The native white Chilean strawberry (*Fragaria chiloensis* spp. *chiloensis* (L.) Mill.) is a perennial herb that was exported from Chile to Europe in the early eighteenth century, and it is the maternal progenitor of the commercial strawberry (*Fragaria x ananassa*). This native species from the Rosaceae family is present in Chile from Libertador Bernardo O’Higgins to the Magallanes region and the Juan Fernández archipelago [20]. This species is a highly used functional food for people in Patagonia for gastrointestinal, blood, gynecological, and obstetric problems [47].

Using a disc diffusion assay, it was shown that *F. chiloensis* leaf methanol extract exhibited an antimicrobial effect against *B. subtilis, Enterobacter aerogenes, E. coli* DC2, *Mycobacterium phlei*, *Pseudomonas aeruginosa* H188, and H187, *Serratia marcescens*, *S. aureus* methicillin-resistant, *S. aureus* methicillin-sensitive and *Salmonella* Typhimurium TA98 [48]. The highest inhibition radium was observed for *E. coli* (20.1–25.0 mm) [48]. The same assay was used to show the antifungal activity of the extract against *Aspergillus flavus, A. fumigatus, Candida albicans, Microsporum cookerii, Microsporum gypseum, Saccharomyces cerevisiae, Trichoderma viridae* and *Trichophyton mentagrophytes*. The highest inhibition radium was observed for *T*. *mentagrophytes* (20.1–25.0 mm) [49].

### 3.8. Geoffroea decorticans

The tree *Geoffroea decorticans* is a native Fabaceae that grows in the forests of the Gran Chaco region in Argentina, the Bolivian Chaco, and in northern Chile from Arica y Parinacota to the Coquimbo region [20]. Fruits contain high levels of sugar, fiber, and a complex mixture of polyphenols that give medicinal properties, such as antinociceptive action, expectorant, and antioxidant activity [33].

Studies have shown the effect of dry extracts from the plant’s bark extracted with methanol against *S. aureus* ATCC 8095, *S. aureus* INEI 2213, *E. faecium* INEI 2464, *P. aeruginosa* ATCC 25617, *S.* Typhimurium INPPAZ 201, *Klebsiella pneumoniae* ATCC 10031 and *E. coli* ATCC 25922. The most effective MIC value was 0.08 mg/mL against *S. aureus* INEI 2213. In addition, 80 mg/mL of the extract inhibited the growth of *S. aureus* liquid culture compared with DMSO solvent. This inhibition could affecting bacterial respiratory activity [50].

Ethanolic extracts from dried leaf and steam and the isolated metabolites from these fractions ((3R)-5,7,2′,3′-tetrahydroxy-4′-methoxy-5′-prenylisoflavanone and (3R)-7-2′-3′-trihydroxy-4′-methoxy-5′-prenylisoflavanone) were analyzed for its antifungal properties against *A. flavus* IEV 018, *A. parasiticus* NRLL22, *A. nomius* 13137 and *A. nomius* VCS23 [51]. Using the radial growth inhibition test, extract (0.4 mg/mL), and prenylsoflavone metabolites (50 μg/mL) diminished radial growth compared to control. Prenylsoflavone compounds had the highest percentage of inhibition against *Aspergillus nomius* 13137. MIC and minimal fungal concentration (MFC) ratios for prenylsoflavone compounds were determined to be between 9 and 21 μg/mL, which were slightly lower than the antifungal clotrimazole (3–10 μg/mL) against the *Aspergillus* strains [51].

### 3.9. Laurelia sempervirens

This endemic Chilean tree belongs to the Monimiaceae family and naturally grows from the Libertador Bernardo O’Higgins to Los Lagos region, usually from the coast up to 1000 masl [20]. As in other family species, *Laurelia sempervirens* leaves are aromatic and can be used as a spice [33], analogous to basil leaves.

Leaves were used to elaborate EOs using hydrodistillation, and GC-MS was used to detect compounds present in the sample, where safrole was the most abundant [52]. In other work, aerial parts of the plant were used to elaborate EOs using a water distillation method. The sample analyzed by GC-MS detected six compounds, with isazafrol being the most abundant [45].

The antimicrobial activity of EOs and purified compounds against *H. pylori*, *S. aureus*, *E. coli,* and *C. albicans* was analyzed using the agar disk diffusion assay. The compound limonene had a MIC of 32 μg/mL against the clinical isolates *H. pylori* and *S. aureus* [45]. An undiluted aliquot of EOs from leaves had a percentage of inhibition of 36% against the plant pathogen fungi *Phragmidium violaceum* [52].

Ethanolic extraction of leaves had an MIA of near 1 mg against *E. coli* EDL 933 and *P. aeruginosa* PAO1. In addition, methanolic steam extraction had MIA of near 1 mg against *S. aureus* ATCC 6538 [29].

### 3.10. Laureliopsis philippiana

*Laureliopsis philippiana* (Looser) R. Schodde (Monimiaceae) is an aromatic tree present in Chile and Argentina, usually from sea level up to 1000 masl [20]. In Chile, it is found mainly as part of the temperate forest but is distributed from Maule to Aysen regions [20]. Leaf infusions can be used to treat the common cold, cough, sore throat, and headaches [33]. Like other Monimiaceae and Lauraceae, it contains EOs with insect-repellent activity [53].

EOs from leaf and bark were extracted by hydrodistillation, and compounds were detected by GC-MS. Six compounds were identified in the leaf and twenty nine compounds in the bark. Safrole and β-phellandrene were most abundant in the leaf. On the other hand, isosafrole, safrole, eucalyptol, methyleugenol, and eugenol were most abundant in the bark [54].

EOs and the isolate compound safrole had MIC values between 50 and 200 μg/mL, tested against shellfish and fish pathogen *Saprolegnia* spp. [54]. Methanolic extraction of leaves had MIC values of less than 1 mg against *E. coli* ATCC 11229, *S. aureus* ATCC 6538, *B. subtilis* ATCC 6633, *S. pneumoniae* (1472-04, 1327-05, 1205-05, and 1322-05 ATCC strains), and MIC of less than 0.5 mg against *P. aeruginosa* ATCC 9027 and MIC less than 0.25 mg against *Penicillium expansum* IMI 285521 [29].

### 3.11. Peumus boldus

*Peumus boldus* Mol. is an aromatic tree that belongs to the Monimiaceae family. The species is endemic to central and central-south Chile [20]. It is a ubiquitous tree of the sclerophyllous forest, typical of the Mediterranean portion of the country. Its fruits are edible and highly aromatic, and their leaves are mostly consumed as an infusion for gastrointestinal and hepatic problems but mixed with "mate" for their flavor and aroma [33].

Aqueous extract from dried leaves exhibited inhibitory activity against *H*. *pylori* J99, a biopsy isolate, and the reference strain *H. pylori* ATCC 43504. Both extract and different chromatographic fractions were assayed to test the MIC using the broth dilution method, reporting a value above 1500 μg/mL. However, urease inhibition was variable, with fraction F5 being the most active with an IC_50_ value of 15.9 gallic acid equivalents/mL. Furthermore, the F5 fraction and extract exerted an anti-adherent effect of *H. pylori* in human gastric adenocarcinoma cells. These two properties of the fraction and extract depended on the presence of polymeric procyanidins [55].

EOs from dried leaves, obtained by hydrodistillation, were subjected to GC-MS analyses obtaining α-pinene, β-pinene, α-terperpine, ρ-cimene, terpinen-4-ol, α-terpinolene, ascaridol, α-gurjunene and espatulenol [56]. EOs at 3000 μg/g produced a negative impact on the growth of *A. flavus* Link (RCD65 and RCI105) and *A. parasiticus* Speare (RCT20 and RCD106) on maize and peanut [56,57].

Dry extracts were obtained with water and analyzed by HPLC-MS, where the following phenolic compounds were identified: catechin, epicatechin, and rutin as the main components [58]. Extracts were tested against *E. coli* ATCC10536, *E. coli* PeruMycA 2, *E. coli* PeruMycA 3, *P. aeruginosa* PeruMycA 5, *S.* Typhi PeruMycA7, *B. cereus* PeruMycA 4, *B. subtilis* PeruMycA 6 and *S. aureus* ATCC 6538, whose MIC were between 24,8 and 198.4 μg/mL; for *E. coli* ATCC10536, the lowest MIC was detected. The same test was applied to dermatophytes and yeast; *C. tropicalis* DBVPG 6184, *C. albicans* DBVPG 6379, *C. parapsilosis* DBVPG 6551, *C. albicans* DBVPG6183, *T. mentagrophytes* CCF 4823, *T. tonsurans* CCF 4834, *T. rubrum* CCF 4879, *T. rubrum* CCF4933, *A. crocatum* CCF 5300, *A. quadrifidum* CCF 5792, *T. erinaceid* CCF 5930, *A. gypseum* CCF 6261, *A. currey* CCF 5207 and *A. insingulare* CCF 5417, where the lowest MIC was for *T. tonsurans* (9.84 μg/mL) [58]. Finally, antimicrobial effects of leaf extracts were also observed against *S. aureus* [59,60].

On the other hand, the aporphine alkaloid boldine found abundantly in the plant’s leaves and bark reduced the growth of the parasite *Trypanosoma cruzi* (Tulahuen strain) on the seventh day of culture using concentration above 500 μM. The same effect was reported for a *T. cruzi* LQ strain and *T. cruzi* DM 28c clone. IC_50_ for the two strains and the clone were 110, 115, and 120 μΜ, respectively [61]. Boldine at concentrations of 100 and 600 μg/mL was also able to reduce the infection to 81 and 96%, respectively, of the amastigotes of *Leishmania amazonensis* in a murine macrophage model [62]. Finally, boldine at 100 and 50 μΜ was able to inhibit the replication of human deficiency virus (HΙV-1_NL4-3_) on CEM-GXR cells and hepatitis C virus (HCV) on Hun-7.5 cells, respectively [63].

### 3.12. Prumnopitys andina

*Prumnopitys andina* (Poepp. ex Endl.) de Laub. is a mountainous evergreen conifer native to south-central Chile [20]. Fruits are edible and can be used for jam and fermented drinks. 

In other work, bark and wood were extracted with methanol and analyzed with different chromatographic methods obtaining diterpenes: abietatriene, 6,7 dehydroferruginol, ferruginol, and honokiol in wood and bark. Meanwhile, abietatriene, acetylferruginol, ferruginol, and isopimarol were found in bark [64]. Furthermore, antibacterial activity was studied for isolated diterpenes in *B. brevis, B. subtilis, E. coli, Micrococcus luteus, Providencia* sp., *Pseudomonas* sp., *Shigella* sp., *S. aureus*, *E. faecalis*, *Streptococcus pyogenes*, where totarol, ferruginol, dehydroferruginol and acetylferruginol evidenced high inhibition ratio [64]. In addition, diterpenes were also tested in *Aspergillus* sp., *Fusarium fujikuroi*, *F. ciliatum*, *Mucor miehei*, *Nematospora coryli*, *P. notatum,* and *Paecilomyces variotii*, where totarol and ferruginol presented high inhibition ratios [64].

Stem bark was extracted sequentially with hexane, chloroform, acetone, and methanol to isolate the abietane diterpene 2β-acetoxyferruginol. This metabolite was tested against *S. aureus* strain ATCC 25923, *S. aureus* clinical isolated XU212 and EMRSA-15, *S. aureus* SA1199B, and *Propionibacterium acnes* ATCC 6919. The MIC of 2β-acetoxyferruginol for these strains was between 4 and above 128 μg/mL, where *P*. *acnes* ATCC 6919 presented the lowest MIC [65].

### 3.13. Quillaja saponaria

*Quillaja saponaria* Mol. is an endemic tree found in the coastal range and the foothills of the Andes in semiarid central Chile up to central-south Chile in the Araucania region [20]. It is a monotypic species from the Quillajaceae family, and its inner bark has long been used for hair and wool washing due to its high content of saponins (detergent-like compounds), where triterpene aglycone quillaic acid is the most common core molecule, which can be glycosylated with glucuronic acid, rhamnose, hexose, and a fatty acyl chain at different ratios at C3 and C28 positions [66].

In a study performed on *E. coli* K-12, this bacterium was exposed to three different commercial saponins and extracted from *Yucca schidigera* (DK Sarsaponin 30) at concentrations of 0.05–1.0% *w/v,* and it was observed a different concentration-dependent response [67]. Growth inhibition was observed on two at a concentration above 0.25% and one at a concentration above 0.5%. In one brand, that growth increase was observed at a concentration above 0.1%. These results suggest different antimicrobial activities dependent on the source of the saponins [67].

Aqueous bark commercial extract, as mentioned above, containing saponins, polyphenols, and tannins, has demonstrated antimicrobial activity against Shiga-toxin (STEC) *E. coli* O157:H7 producers strains (ATCC 43894, ATCC 43888, Jack in the box and CIDER outbreaks), human pathogens responsible for outbreaks of bloody diarrhea and hemolytic uremic syndrome worldwide, as well as against non-O157 STEC *E. coli* strains (O145:H18, O26:H11, O121:H19, O103:H11, O45:H2, and O111:H8). After 1 h at 37 °C of treatment with extract, bacteria were undetectable in tryptic soy agar. Furthermore, scanning electron microscopy showed that treated bacteria exhibited damage in membranes [68].

A saponin-rich extract containing 8.0–10% saponin was active against *S. aureus* ATCC 49525, *S.* Typhimurium NCIM 2719, and *E. coli* ATCC 933 with a MIC of 0.1 mg/mL [69].

Aqueous extracts less than 1.0 mg/mL exhibited antiviral activity against viruses like vaccinia virus, herpes simplex virus type 1, varicella-zoster virus, human immunodeficiency viruses 1 and 2, rotavirus, and reovirus, by blocking the virus-host attachment [70,71,72].

### 3.14. Schinus polygama

*Schinus polygama* (Cav.) Cabrera is a shrub or small tree of about 1.2–2 m in height from the Anacardiaceae family. In Chile, it grows from Arica and the Parinacota Region (north of Chile) to the Araucania Region (central Chile), and it is also naturally present in Argentina and Bolivia [20]. It is currently naturalized in California, known as Hardee peppertree or Chilean peppertree. Its fruits are edible fresh, and the latex that forms the cortex is used to treat cataracts, skin rashes, and even clean wounds [33].

Dried leaves from the plant were recollected in Argentina and were hydro-distilled to obtain a 0.2% EOs solution and later on tested on paper disc diffusion against bacteria *B. cereus* and the yeast *C. albicans* showing a MIC of 56.2 of 237 μg/mL, respectively. The composition of the extract was analyzed by GC-MS, identifying around thirty compounds, including monoterpenes and sesquiterpenes, where the molecules α-phellandrene and limonene were significant components [73].

EOs extracted by hydrodistillation from leaves and fruits were used to analyze composition by GC-MS. Thirty eight compounds were detected, including camphene, camphor, endobornyl acetate, veridiflorol, and longifolol that were exclusively found in fruits. Meanwhile, β-myrcene, transgeraniol, β-cubebene, aromadendrene, spathulenol, epoxy-alloaromadendrene, and α-cadinol were exclusively found in leaves and fruits, where the most abundant was β-pinene [74]. In addition, EOs and a dichloromethane extract were tested using bioautography agar overlay against *E. coli* (ATCC 8739), *Klebsiella pneumoniae* (isolated from a patient), *S. aviatum* (ATCC 2228), *S. aeruginosa* (ATCC 14207), *S. aureus* (ATCC 6538P), *Micrococcus flavus* (ATCC 10290), *B. subtilis* (ATCC 6633) [74]. MIC for EOs from leaves was evaluated against *S. aureus, B. subtilis,* and *Micrococcus flavus.* The values were 140, 10, and 13 μg/mL, respectively [74].

### 3.15. Ugni molinae

*Ugni molinae* Turcz. is a small evergreen shrub from the Myrtaceae family, native to Chile and Argentina, which grows in the Coastal and Andes Mountain ranges in the south-central region of Chile [20]. It is very aromatic (typical of the family) with edible reddish fruits rich in antioxidants and antimicrobial properties [75]. *U. molinae* has also been used to treat some infections and diverse types of pains [33]. It is commercially produced by New Zealand as New Zealand cranberry, and to a lesser extent, in Chile; however, evidence suggests that domestication alters its secondary metabolism [76].

Ethanolic and methanol acid extracts of *U. molinae* fruits were evaluated through liquid chromatography and mass spectrometry. Ethanolic extracts showed the presence of three major compounds; caffeic acid 3-glucoside, quercetin-3-glucoside, and quercetin, while in the methanolic acid extract, the major compounds were cyanidin-3-glucoside, pelargonidin-3-arabinose and delphinidin-3-glucoside [77]. In addition, different volumes of these extracts were also tested against pathogens *E. coli* ATCC 25922 and *S. enterica* serovar Typhi ATCC 14028, which are common food contaminants. Both extracts presented activity except ethanolic extract on *S. enterica* [77].

Shene et al. (2009) studied the plant’s composition of leaves and fruits extracted from three locations in Chile (mountain, coast, and valley) using HPLC. Analysis of fruit 50/50 (water/ethanol) mountain sample identified the presence of myricetin glucoside, quercetin dirhamnoside, quercetin glucoside, and quercetin glucuronide. On the other hand, analysis of leaves 50/50 mountain sample identified myricetin glucoside, quercetin dirhamnoside, quercetin glucoside, quercetin glucuronide, myricetin xyloside, quercetin rhamnoside and flavan-3-ols in monomeric and in polymeric forms. Furthermore, the antimicrobial activity of the extract from fruits prepared at different ratios of water/ethanol was analyzed against pathogenic *S. aureus* ATCC 25923. Interestingly, 100% ethanol extraction showed no effect on antimicrobial activity, manifested when water is added to alcohol, suggesting the presence of active molecules soluble in water. For all the three locations, the 50/50 ratio has the highest inhibition zone [78]. Similar results were obtained for leaf extracts against *S. aureus* ATCC 25923 and other two pathogens, *K. pneumoniae* ATCC 13883 and *P. aeruginosa* ATCC 27853 [78].

Another study identified the phenolic molecules of leave and fruit, where it was possible to identify catechin hydrate, rutin, isoquercitrin, ellagic acid dihydrate, quercitrin hydrate, and isorhamnetin-3-*O*-glucoside [75]. The antimicrobial effects of fruits and leaves of *U. molinae* were determined by measuring MIC and MBC against *E. coli* CECT 434 (ATCC 25922) and *L. monocytogenes* CECT 934 (ATCC 19114). The 50/50 (ethanol/water) mixture of leaves extracts presented the highest MIC and MBC against *L. monocytogenes* with a value of 0.07 and 0.09 mg/mL, respectively [75]. The antimicrobial activity was observed against the plant pathogen *Penicillium expansum* using both extracts, showing an inhibition growth of 3.79 and 4.76%, respectively; however, it was considered low for this kind of plant.

Using the TLC agar overlay protocol, an MIA of ~1 mg against *P. aeruginosa* PAO1 of a methanolic stem extract was observed [29].

## 4. Plant Metabolites Incorporated in Films and its Application in Food Industry

In the last decade, research into packaging with bioactive ingredients has incorporated significant advances, improving essential characteristics such as protection and food preservation [23]. New materials for food packaging have been developed to meet the requirements of fewer additives, biodegradability, and low risk to human health. The primary role of food packaging is food product protection from the external environment. Hence, its goal is to keep food economically while fulfilling consumer and industrial requirements, minimizing environmental effects. Additionally, bioactive packaging might contain antimicrobial components, which interact with biological molecules and may restrict the growth of microorganisms [79]. Concerns about chemoactive packaging led to new alternatives like incorporating bioactive compounds from natural sources [80]. Because of the growing consumer demand for natural products, synthetic compounds are replaced by natural molecules such as EOs, polyphenols, and other natural extracts [81]. The films used in these active packages can be biopolymers, plastics, or other polymers to which plant extract or active molecules with antimicrobial activity can be added [82].

There are few studies on AP using plant extracts from Chilean species. For instance, Genskowsky et al. (2015) reported antibacterial and antioxidant properties in a chitosan film with added *A. chilensis* extract. This mixture was dose-dependent against *Listeria innocua, Serratia marcescens, Aeromonas hydrophila, Achromobacter denitrificans, Alcaligenes faecalis, Pseudomonas fluorescens, Citrobacter freundii,* and *Shewanella putrefaciens* [83]. Thus, it is a good plant, particularly its edible berries, to be used for AP in the food industry. Microencapsulation of *P. boldus* EOs by complex coacervation method was used to control microbial content of in-pod stored peanut after five months. A significant reduction of fungal content was observed [84,85]. Antioxidant and bacteriostatic effects were observed when *Q. saponaria* polyphenols were added to the meat or chicken marination process at 6 °C. The application of polyphenols reduced the aerobic mesophiles and total coliform count by at least one order of magnitude [86]. Finally, there is evidence of antimicrobial activity in an AP made of a polyethylene film coated with a methylcellulose layer with added *U. molinae* leaf extract [87]. The packaging conserved its antimicrobial effect against *Listeria innocua* for at least 60 days and did not alter the organoleptic properties of the food inside. Likewise, extracts of other Chilean native or endemic plants with a high metabolite content, like those mentioned in this review, could be used in creating new AP with antimicrobial activity, but further research in this area is required.

EOs consist of liquids extracted from diverse parts of aromatic plants like barks, seeds, flowers, peel, fruit, roots, leaves, wood, fruits, and whole plants [88]. They are typically obtained by steam distillation, mechanical processes from the epicarp of citrus fruits, dry distillation, and solvent extraction [89]. Azadbakht et al. (2018) examined the incorporation of *Eucalyptus globulus* EOs in chitosan polymers and studied its antimicrobial activity. Results showed that increasing EOs concentration could improve the reduction value [90]. Moreover, Lin et al. (2018) examined the effectiveness of active packaging containing thyme EO/β-cyclodextrin ε-polylysine nanoparticles. Results showed that this nanoparticle incorporated into gelatin nanofibers significantly improved its antimicrobial properties against *Campylobacter jejuni* [91]. In other work, it was evidenced that neem oil from *Azadarichta indica* significantly improved the antibacterial activity in poly (ethylene terephthalate) polyester [92]. EOs of *Ziziphora clinopodioides* and sesame oil in chitosan-linseed mucilage have an antibacterial effect against *L. monocytogenes*, *S. aureus*, *S.* Typhimurium, and *E. coli* in raw rainbow trout fillets [93]. Orsuwan & Sothornvit (2018) formulated a packaging based on active banana flour plasticized with garlic EOs to preserve peanuts and oily food products with complete inhibition of *Aspergillus flavus* growth [94].

Polyphenolic compounds, a particular group of phenolic compounds, have been extensively studied due to their antioxidant and antimicrobial properties. These compounds in the form of extracts or a combination of them have been incorporated into films to avoid the growth of microorganisms. Jang et al. (2011) developed a film constituted by a rapeseed protein–gelatin and grapefruit seed extract. The new film was able to control *E. coli* O157:H7 and *L. monocytogenes* pathogenic bacteria and was tested to control bacteria and fungi present in strawberries [95]. *Gelidium corneum*–gelatin films and grape fruit extract or green tea extracts were elaborated to control microbes from pork loins. In this regard, films could diminish the presence of pre-inoculated *E. coli* O157:H7 and *L. monocytogenes* pathogens [96]. In other work, using the colony count method, films composed of agar/alginate/collagen hydrogels and grape fruit extracts could diminish the presence of the same pathogens [97]. Treatment with UV-A light and curcumin edible coating (cassava-gelatin hydrogel) could reduce *L. innocua* load present in coated sausages, including at 4 °C, which suggests its application for refrigerated food [98]. Sun et al. (2017) prepared a film made of chitosan and young apple polyphenol extract that presented antimicrobial activity against *E. coli*, *L. monocytogenes*. *S. aureus*, yeast, and molds [99]. The same polymer was used with a turmeric extract and tested against *Salmonella* and *S. aureus* [100]. *S. aureus, L.monocytogenes,* and *E. coli* were also sensible in the presence of a film composed of *Punica granatum* L. (pomegranate) peel powder and fish gelatin film-forming solution [101]. Gallic acid incorporated in zein films also had antimicrobial properties against *L. monocytogenes* and *C. jejuni* [102]. Finally, alginate films, green tea extract, or grape seed extract had activity against murine norovirus and hepatitis A [103].

Yao et al. (2020) have developed an antioxidant, antimicrobial, and ammonia-sensitive packaging by adding *Opuntia ficus*-*indica* extract in a mixture of quaternary ammonium chitosan with polyvinyl alcohol with activity against food pathogens such as *S. aureus*, *L monocytogenes,* and *S.* Typhimurium [104]. Likewise, Kanatt et al. (2020) generated a smart active food packaging film containing *Amaranthus* leaf extract to extend the shelf life of chicken and fish during refrigerated storage by acting against *S. aureus*, *B. cereus*, *E. coli,* and *P. fluorescence* [105]. On the other hand, Figueroa-Lopez et al. (2018) studied containers with films based on gelatin and microcrystalline cellulose with *Piper nigrum* oleoresin, achieving inhibition against *S. aureus* and *E. coli* in bread for nine days [106].

Extracts containing bioactive molecules can sometimes degrade with temperature, oxidize, or change the food’s smell and/or taste inside the packaging. Adding oligosaccharides, such as cyclodextrins, can improve the effectiveness of the active plant molecules in the films [107]. For example, a starch-based active packaging with antimicrobial properties can be made by adding an *Acca sellowiana* (Feijoa) by-product [108]. However, as little as 3% of the by-product had a relatively high antimicrobial effect, possibly due to the antioxidants and alkaloids present in the peel [109]. The antimicrobial activity of plant extracts as an additive in AP depends on its chemical composition, which ultimately depends on the plant species and extraction method. It is, however, commonly due to the presence of phenolic compounds such as catechin and thymol [110] and alkaloids, as mentioned above.

## 5. Discussion

This work studied Chilean native and endemic plants regarding their antimicrobial properties. In addition, the antimicrobial plant extract using different extraction methods and its application using different assay methods were also reviewed. In this regard, few studies have attempted to reveal the composition of these extracts and the purification of the metabolites responsible for the antimicrobial activity. A summary of these results and a possible application of extracts on films for antimicrobial control are shown in Table 1.

Although antimicrobial properties have been reported in several plants [111,112,113], the search for these properties in native endemic plants from Chile is relatively scarce. At first glance, few works reported where the responsible(s) molecule(s) for the antimicrobial activity was or were identified. We found that the search for these types of antimicrobial properties is described mainly using extracts without purification of the active molecule(s). If the molecule is purified, studies of structure-function to determine the putative target(s) on microbial cells to determine its mechanism of action are required. There are different mechanisms by which plant metabolites can have an antibacterial effect. Metabolites could present bactericidal, bacteriolytic, or bacteriostatic activities. Bacteriostatic metabolites are generally defined as compounds that inhibit some critical biochemical processes reversibly; bactericidal agents strongly and irreversibly target critical processes, leading to cell death; and bacteriolytic metabolites destroy the cell by lysis, releasing cytoplasmic components [114]. Since several types of molecules compose the extracts, the final effect could be due to single molecules or a combination of them, resulting in a synergic effect [115]. The antimicrobial activity of these native Chilean plants was demonstrated in bacteria, fungi, protozoa, and viruses. Procedures for obtaining the MIC or the MBC could vary depending on the application of distinct methodologies. Then, more work is needed to validate these results using the same procedure (i.e., microplate dilution, disc assay, broth dilution method, etc.).

As was reviewed, metabolites with antimicrobial activity found in Chilean native plants can be classified into EOs, phenolic compounds, alkaloids, and saponins.

EOs are composed of low molecular weight volatiles such as terpenes, terpenoids, aromatic and aliphatic compounds, among others [116]. The mechanisms of action of EOs on bacteria have been reported, and these include degradation of the cell wall, damaging the cytoplasmic membrane, cytoplasm coagulation, damage of membrane proteins, reduction of proton motive force; reduction of intracellular ATP pool, inhibition of quorum sensing (QS) and alteration of cell division [116,117,118,119]. In addition, examples of antibacterial activities have been reported; for instance, Kim et al. (2002) reported the effect of 11 EOs components against different bacteria (*E. coli* O157:H7, *S.* Typhimurium, *L. monocytogenes,* and *Vibrio vulnificus*), where carvacrol had an MBC of 250 μg/mL against *S.* Typhimurium and *V. vulnificus.* Other tested compounds were citral, perillaldehide, terpineol, geraniol, citronellal, and linallol [120]. Interestingly, as mentioned, bacterial QS also is affected by EOs of *Salvia rosmarinus, Syzygium aromaticum*, and *Lavandula angustifolia,* among others [118]. EOs are proposed to be used as food preservatives. However, they seem less active than single molecules and provoke changes in the organoleptic properties of food [119].

Alkaloids belong to a diverse group of molecules containing nitrogen. Some alkaloids were described to present antimicrobial activities against Gram-positive and Gram-negative bacteria, including mycobacteria [121]. Regarding mechanisms of alkaloids, some of them inhibit nucleic acid biosynthesis and cell division, leading to membrane integrity disruption. However, some cases seem to be species-dependent [121]. The alkaloid prosopilosidine, isolated from *Prosopis glandulosa* var. *glandulosa*, was effective against the yeast *Cryptococcus neoformans*, the fungi *A. fumigatus*, and the bacteria *S. aureus* and *Mycobacterium intracellulare* with IC_50_ values of 0.4, 3.0, 0.35 and 0.9 μg/mL, respectively [122]. From *Clausena anisate*, the compound 1-hydroxy-6-methoxy-3-methylcarbazole, also known as clausenol, was found to be active against *E. coli* ST 203, *B. subtilis* ST204, *S.* Typhi ST288, *P. aeruginosa* ST243, *S. aureus* MC27927, *C. albicans* ST388, and *T. rubrum*, having MIC of 7, 14, 12, 14, 1, 3, 5 and 2 MIC μg/mL, respectively [123]. The benzophenanthridine alkaloid chelerythrine from *Zanthoxylum rhoifolium* had a wide range of action against Gram-positive (*S. epidermis*, *S. aureus,* and *B. subitilis*), Gram-negative (*K. pneumonia*, *E. coli*, *P*. *aeruginosa*, *Shigella sonnei*) and yeast (*Candida albicans*, *S. cerevisiae,* and *Cryptococcus neoformans*) using 0.15–12.5 μg of each purified alkaloid from steam bark [124]. From *Zanthoxylum* *tetraspermum,* two alkaloids were obtained, namely 8-acetonyldihydronitidine and 8-acetonyldihydroavicine, which were active against *S. aureus* with a MIC of 1.56 and 3.12 μg/mL, respectively. The former was also active against *Cladosporium cladosporioides* [125]. In *Hosta plantaginea,* the alkaloid 7-deoxy-trans-dihydronarciclasine has been shown to present properties against the tobacco mosaic virus with an IC_50_ of 1.80 μM [126].

Phenolic compounds are grouped into phenols, phenolic acids, flavonoids, xanthones, stilbenes, and lignans. Phenolic compounds have generally demonstrated permeative action by destabilizing the outer membrane, inhibiting enzymes and toxins, and suppressing bacterial biofilm formation, among other effects [127]. Phenolic compounds present in different fruits (catechin, ferulic, gallic, protocatechuic, and vanillic acids) were evaluated against the pathogens *E. coli* 0157:H7 ATCC 43890 and *S. enterica* serovar Typhimurium ATCC 14028, obtaining MIC and MBC of 15–20 mM and 20–30 mM, respectively [128]. In addition, methanol extracts from the peel from *P. granatum* flour were obtained, and eight phenolic compounds were reported, including punicagalin and ellagic acid. MIC and MBC were obtained against different pathogenic bacteria. Nevertheless, the highest inhibitory effects were observed in *L. innocua* (MIC: 20 mg/mL and MBC: 30 mg/mL) [129]. Antimicrobial activity was also observed in a methanol extract from *P. granatum* L. against *S. enteritidis* with a MIC of 4 mg/mL [130].

Finally, saponins can be found in *Quillaja* spp. (*Q. saponaria* and *Q. brasilensis*) and in more than one hundred plant families. These molecules are composed of aglycone core and carbohydrate side chains that present detergent-like properties that have been exploited commercially [131]. This type of molecule’s action mechanism seems to alter the bacterial cell membrane since quillaja extracts are involved in pore formation, promoting leakage of cytoplasm components [132]. However, mainly due to the different composition of molecules in the extract seems to be challenging to describe antimicrobial properties. In some cases, saponin at a dose of 12 μg/mL enhances *E. coli* strains growth [133]. Around sixty different types of saponins exist in *Q. saponaria* [131].

## 6. Conclusions

Some Chilean plant species present antimicrobial activities that can be observed by extraction with different solvents and vary depending on the plant tissue. Thus, the highly endemic Chilean flora presents a great potential to develop new antimicrobials, urgently needed due to the increased resistance of microbes to existing antimicrobial compounds. Antimicrobial activities in these plants have been observed for some Gram-positive bacteria, Gram-negative bacteria, fungi, yeast, protozoa, and viruses. Scientific validation of the plant metabolites as antimicrobials has been mainly explored in vitro. Their effects in active films and clinical studies are needed to properly validate their use in the food industry to control food pathogens on AP. Existing data suggest that at least some Chilean plant species can be used to elaborate active films, maintaining their antimicrobial properties without changing the organoleptic properties of food. This should encourage further research into this topic, primarily focusing on endemic species and/or those with known traditional use as medicinal plants. Further purification of metabolites to single molecules is also needed to establish concrete activities and study putative microbial cellular targets. This information could help understand whether the biological effect is due to single, additive, or synergic effects of antimicrobial metabolites. Lastly, still little is known about Chilean native and endemic plants, with yet many species that have not been phytochemically studied. Therefore, more studies are needed to explore Chilean plant biodiversity regarding its chemical components and biological activity to find new compounds for food preservation.

## Figures and Tables

**Table 1:** Summary of endemic/native plants from Chile involved in antimicrobial activities and their application in food packaging. Information on the polymer used and the proven effect of active films made with extract or chemicals from those species is included when available. NDA: no available data.

Plant	Target Microorganism	Polymer Used	Reported Effect/Possible Application	References
*Acaena magallanica*	*Escherichia coli, Microsporum canis*	NDA	NDA	[25]
*Aristotelia chilensis*	*Escherichia coli*, *Staphylococcus aureus*, *Bacillus subtilis* *Listeria innocua*, *Serratia marcescens*, *Aeromonas hydrophila*, *Achromobacter denitrificans*, *Alcaligenes faecalis*, *Enterobacter gergoviae*, *Enterobacter amnigenus, Shewanella putrefaciens*	Chitosan	Antimicrobial effect against *Aeromonas hydrophila*, *Achromobacter denitrificans*, *Alcaligenes faecalis*, *Citrobacter freundii*, *Listeria innocua*, *Pseudomonas fluorescens*, *Serratia marcescens*, and *Shewanella putrefaciens*/active packaging	[28,29,79]
*Azorela acaulis*	*Mycobacterium tuberculosis* *Trichomonas vaginalis*	NDA	NDA	[31,32]
*Berberis microphylla*	*Bacillus cereus*, *Staphylococcus aureus*, *Staphylococcus epidermidis*, *Bacillus subtilis* *Staphyloccocus aureus*, *Escherichia coli* *Trypanosoma cruzi*	NDA	NDA	[28,37,39]
*Buddleja globosa*	*Staphylococcus aureus*, *Escherichia coli* *Paenibacillus larvae* *Trichophyton rubrum*, *Tricophyton interdigitale*, *Epidermophyton floccosum* *Plasmodium falciparum*	NDA	NDA	[41,42,43,44]
*Cryptocarya alba*	*Trypanosoma cruzi*, *Leishmania* spp. *Helicobacter pylori*, *Staphylococcus aureus*, *Escherichia coli*, *Candida albicans*	NDA	NDA	[45,46]
*Fragaria chiloensis* spp. *chiloensis*	*Aspergillus flavus*, *Aspergillus fumigatus*, *Candida albicans*, *Fusarium tricuitum*, *Candida albicans*, *Fusarium tricuictum*, *Microsporum cookerii*, *Microsporum gypseum*, *Saccharomyces cerevisiae*, *Trichoderma viridae*, *Trichophyton mentagrophytes* *Bacillus subtilis*, *Enterobacter aerogenes*, *Escherichia coli*, *Mycobacterium phlei*, *Pseudomonas aeruginosa*, *Serratia marcescens*, *Staphylococcus aureus*, *Salmonella* Typhimurium	NDA	NDA	[48,49]
*Geoffroea decorticans*	*Staphylococcus aureus*, *Escherichia coli*, *Klebsiella pneumoniae*, *Pseudomonas aeruginosa*, *Salmonella* Typhimurium, *Enterococcus faecium*, *Staphylococcus aureus* *Aspergillus flavus*, *Aspergillus parasiticus*, *Aspergillus nomius*, *Aspergillus nomius*	NDA	NDA	[50,51]
*Laurelia sempervirens*	*Phragmidium violaceum* *Helicobacter pylori*, *Staphylococcus aureus*, *Escherichia coli*, *Candida albicans*	NDA	NDA	[29,45,52]
*Laureliopsis philippiana*	*Saprolegnia* spp. *Escherichia coli*, *Staphylococcus aereus*, *Bacillus subtilis*, *Streptococcus pneumoniae*, *Pseudomonas aeruginose*, *Penicillium expansum*	NDA	NDA	[29,54]
*Peumus boldus*	*Helicobacter pylori* *Aspergillus flavus*, *Aspergillus parasiticus* *Escherichia coli*, *Pseudomonas aeruginosa*, *Salmonella typhy*, *Bacillus cereus*, *Bacillus subtilis*, *Staphylococcus aureus*, *Candida tropicalis*, *Candida albicans*, *Candida parapsilosis*, *Trichophyton rubrum*, *Trichophyton tonsurans*, *Trichophyton erinacei*, *Arthroderma crocatum*, *Arthroderma quadrifidum*, *Arthroderma gypseum*, *Arthroderma currey* and *Arthroderma insingulare* *Staphylococcus aureus* *Trypanosoma cruzi* *Leishmania amazonensis* Human immunodeficiency virus 1 and Hepatitis C virus	Gelatin and gum arabic	Antimicrobial effect against *Aspergillus* and *Penicillium* spp./active packaging	[55,56,57,58,59,60,61,63,80,81]
*Prumnopitys andina*	*Bacillus brevis*, *B. subtilis*, *E. coli*, *Micrococcus luteus*, *Providencia* sp., *Pseudomonas* sp., *Shigella* sp., *Staphylococcus aureus*, *Enterococcus faecalis*, *Streptococcus pyogenes* *Aspergillus* sp., *Fusarium fujikuroi*, *F. ciliatum*, *Mucor miehei*, *Nematospora*, *Propionobacterium acnes*	NDA	NDA	[64,65]
*Quillaja saponaria*	*Escherichia coli* *Escherichia coli* *Escherichia coli*, *Staphylococcus aureus*, *Salmonella typhimurium* Rotavirus, vaccinia virus, herpes simplex virus, varicella-zoster virus, human immunodeficiency virus	NDA	Antimicrobial effect against mesophilic aerobe and total coliform/chicken marination	[68,69,70,71,72,82]
*Schinus polygamus*	*Bacillus cereus*, *Candida albicans* *Escherichia coli*, *Klebsiella pneumoniae*, *Salmonella aviatum*, *Salmonella aeruginosa*, *Staphylococcus aureus*, *Micrococcus flavus* and *Bacillus subtilis*	NDA	NDA	[73,74]
*Ugni molinae*	*Escherichia coli*, *Salmonella enterica*, *Staphylococcus aureus* *S. aureus*, *K. pneumonia* and *P. aeruginose* *Escherichia coli*, *Listeria monocytogenes* *Penicillium expansum* *Pseudomonas aeruginosa*	Polyethylene film/methylcellulose	Antimicrobial effect against *Listeria innocua*/active packaging	[29,75,77,78,83]

## Data Availability

Not applicable.
